# Noise Annoyance in the UAE: A Twitter Case Study via a Data-Mining Approach

**DOI:** 10.3390/ijerph18042198

**Published:** 2021-02-23

**Authors:** Andrew Peplow, Justin Thomas, Aamna AlShehhi

**Affiliations:** 1 Division of Engineering Acoustics, Department of Construction Sciences, Lund University, 221 00 Lund, Sweden; 2 College of Natural and Health Sciences, Zayed University, Abu Dhabi 144534, United Arab Emirates; justin.thomas@zu.ac.ae; 3 Electrical and Computer Engineering, Khalifa University, Abu Dhabi 127788, United Arab Emirates; aamna.alshehhi@ku.ac.ae

**Keywords:** Twitter, noise, annoyance, geolocation, noise classification

## Abstract

Noise pollution is a growing global public health concern. Among other issues, it has been linked with sleep disturbance, hearing functionality, increased blood pressure and heart disease. Individuals are increasingly using social media to express complaints and concerns about problematic noise sources. This behavior—using social media to post noise-related concerns—might help us better identify troublesome noise pollution hotspots, thereby enabling us to take corrective action. The present work is a concept case study exploring the use of social media data as a means of identifying and monitoring noise annoyance across the United Arab Emirates (UAE). We explored an extract of Twitter data for the UAE, comprising over eight million messages (tweets) sent during 2015. We employed a search algorithm to identify tweets concerned with noise annoyance and, where possible, we also extracted the exact location via Global Positioning System (GPS) coordinates) associated with specific messages/complaints. The identified noise complaints were organized in a digital database and analyzed according to three criteria: first, the main types of the noise source (music, human factors, transport infrastructures); second, exterior or interior noise source and finally, date and time of the report, with the location of the Twitter user. This study supports the idea that lexicon-based analyses of large social media datasets may prove to be a useful adjunct or as a complement to existing noise pollution identification and surveillance strategies.

## 1. Introduction

One of the striking technological patterns emerging at the end of the last century was the fast development and production of advanced devices (personal computers, smartphones and so on) across varying backgrounds [1]. One result of these improvements is the ascent of progressively enormous datasets, seemingly across all socioeconomic sectors. These datasets are often alluded to as “big data”, a term generally utilized concerning the volume (speed of development) and an assortment of information. Beyond volume, velocity and value, commentators have also referred to the potential value attached to these datasets [1]. Value is the idea that industrially and culturally important data can possibly be utilized from these datasets for academic or commercial purposes. For example, Google search query data have been used for monitoring influenza outbreaks. Using the geolocation of search inquires, the spread of an outbreak can be monitored with greater speed and accuracy than conventional epidemiological surveillance techniques [2,3]. Psychological constructs, such as happiness or subjective wellbeing, have also been studied [4,5,6], self-concept [7], religiosity [8] and the use of profanity [9].

In the present study, we use similar big data analytic techniques to introduce a method for estimating both the prevalence and location of noise annoyance. In addition, the location and the number or tally of the tweet activity can be stored compared to frequency and geolocation, as discussed in the article [2]. In the last five years, the earliest known analytical methods for studying responses to sound from digital media was performed by the authors [10], who based their work on data from a repository of audio samples in Chatty Maps centered within London and Barcelona. The authors were successful in tagging and locating noise events and thus was able to create a large taxonomy and a lexicon reflecting both negative and positive aspects characterized into 6-noise sources; mechanical, transport, nature, human, music and indoor. This classification was also used in this work and by the authors [11,12], where the focus of their in-depth studies was based on noise response through social media, where a subset of Twitter responses (tweets) was analyzed in London, UK. The latter study grouped geographic areas into socioeconomics groups and was able to extract responses in terms of correlations to hypertension, which shows, above all, that meaningful conclusions can be drawn from these studies, which can benefit not only urban planners but also stakeholders in medical policies.

Noise is a growing public health concern, linked with issues that severely affect hearing, sleep and attribute to hypertension and heart disease. Individuals are increasingly using social media to voice complaints about problematic noise sources. This behavior—using social media to post noise-related complaints and comments—might help us better identify troublesome hotspots and take corrective action. This article is a concept study, which manages an examination of tweeted noise complaints sent from within the United Arab Emirates (UAE) during 2015. Such reports have been organized in an information base and grouped by (1) common types of noise sources (human factors, music, construction, traffic), (2) exterior or interior noise source (domestic, industrial, others such as ventilation noise) and (3) data and exact time of report with the location of the receptor/Twitter user.

A 2016 European study found that people living next to noisy roads were 25% more likely to have symptoms of depression than people in quieter areas, even when adjusting for socioeconomic factors, [13]. Noise criteria, for health reasons, are governed by sound energy levels averaged over a certain time period. The period is normally the 24 h cycle, which is divided into day/evening/nighttime (07:00–19:00–23:00–07:00) with weightings emphasizing the evening and nighttime levels. In 1995 the World Health Organization (WHO) declared, “*The main negative effects of such noise on people are disturbances of communication, rest and sleep, and general annoyance. Over long periods of time, these effects have a detrimental influence on wellbeing and perceived quality of life.*” [14].

Annoyance or irritation are commonly reported responses to ambient or environmental noise. Arising from non-positive effects on daily routines, thoughts, feelings, sleep deprivation, or daily rest can lead to negative emotions, such as distress, exhaustion, and other stressors [15,16,17]. Hence, this study is focused on the subjective response to noise, reporting annoyance by tweets. This has the advantage that the study is ecologically valid, capturing real-time complaints without any of the response biases or reactivity that can be associated with traditional self-report survey methodologies.

Noise pollution represents a complex issue in the evaluation of life equality, especially in built-up zones. Noise is defined as unwanted sound; it is the characteristic physical nature of sound that can transmit in the air and through building structures that represent both the level and character (for example, low-frequency sound through wall partitions) of noise annoyance. These sounds emanate from both predictable (traffic) or unpredictable (neighborhood) noise sources. Adjustment for bias, confounders, socioeconomic status (SES) and lifestyle habits are important factors to consider in scientifically controlled assessments on the impact of noise [18,19]. For example, in work in [20], the author has shown the negative impact on property prices due to traffic noise—these results can readily skew the response to a controlled questionnaire. It is therefore important to recognize the difficulty in considering lifestyle or biased opinion in scientific surveys.

In the WHO report, it was established that twice as many city-dwellers (23%) are reported as having suffered from noise compared to those living in rural settings (10%). The document detailed reports largely from street or neighborhood noise, but nevertheless, the difference in numbers come as no surprise

The use of the Twitter dataset for quantifying noise disturbance will be enhanced by the availability of Geographical Positioning System (GPS) location data, as well as the day and actual instantaneous time in which the subject reported the “annoyance”. This is valuable data, which has not been reported previously. The main aim of this cross-sectional pilot study is to assess the subjective noise annoyance and disturbance among population groups in or surrounding built apartments or villas situated within the emirates of UAE. To begin to comprehend the actual perception of unwanted sounds by residents, we present the analysis of reported complaints of noise pollution registered in the United Arab Emirates (UAE) via tweets. This methodology was implemented via open-access Python language, which has the capability to “tag” noise complaints via location, which could also be implemented as “Live” monitoring.

## 2. Methodology

The data used in this study was a randomly extracted subset of the UAE Twitter data for 2015 using their Historical PowerTrack enterprise product, although it should be possible to return similar results from a Twitter API. The dataset comprised 8.2 million tweets—approximately 10% of the total number of tweets that year—collected between 1 January 2015 to 31 December 2015. Provided by Twitter, the company, the material included as part of a large data download service established to support research. The data obtained included fields related to the user and fields related to the text (tweet). The data were collected via a Query search and coded using Python/Anaconda. This allowed the body text of each tweet to be subjected to query criteria, such as body text, which contains any one of the keywords from a chosen lexicon. The dataset was explored to check if it corresponded to expected national norms, for example, the percentage of tweets per emirate, the rate of Arabic use by Emirates. The data confirmed all expectations. Using a subset is, however, a limitation, and future studies should use larger datasets, ideally comprising the whole corpus of tweets for the region and timeframe under exploration. User features included display name and user description. Text level features included text language, geolocation, location name, and posted time. There are 24 different languages; 44% of tweets were Arabic and 39% English. For the purpose of this case study, we were limited to exploring English tweets only. Table 1 summarizes additional information concerning the data set.

Although the objectives of this article are factors involved in the study of “tweeting” the user’s annoyance of noise, it is important to consider the layout of the data collected. The size of data, “participation patterns”, and coverage, with details on individual cases and more specific patterns, are covered. To avoid missing descriptors, we identified words in British-English and American-English using parentheses and included many versions of words such as “noise” or “noisy” by wild-card descriptors, (*). We also decided against adopting the Arabic language due to complications; the lexicon, Table 2, was used to filter the data from which a total of 272 tweets were identified. The number of “hits” we were able to establish as related to a noise incident was crucially determined by the wording in the lexicon. After many attempts, convergence was not always certain; we decided on the lexicon shown below. Convergence here is meant in the sense of convergence in a reasonable time. This was not performed in a truly scientific manner but should be designed with more diligence in further attempts. Basically, we found the lexicon we used to provide the most efficient number of useful “hits”. However, most of, which were false-positives, for example, “*Sleeping at Last’s music is phenomenally, sensationally, and truly beautiful*.” was sent on 18 April 2015. However, one example of an annoyed tweeter, “*Hey ya, construction noise from the site between Mag218 & 23 Marina is to [sic] loud* “, tweeted on 5 May 2015, was included. Manually removing these false positives reduced the dataset to 38 tweets positively identified as strongly correlated to the sender’s annoyance. Data for the years 2016 and 2017 were available to the authors, but the material was incomplete or only partially available in some areas. To determine any trends over a full 12 month period, we decided to use the 2015 data exclusively.

## 3. Results of Case Study

**Figure 1:**
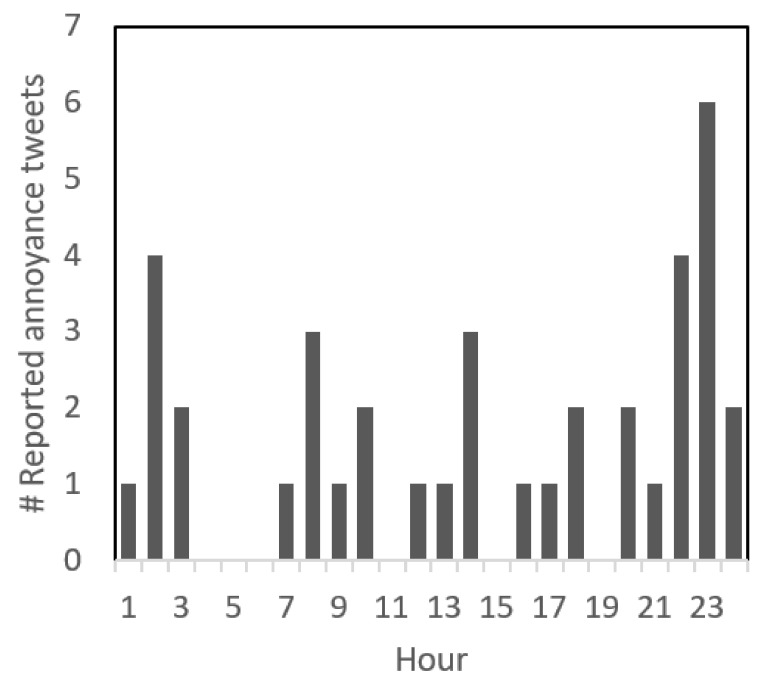
Frequency and time at which tweets were reported. Representing hourly intervals, the *x*-axis represents the 24 h period, i.e., midnight-to-midnight.

Sustained exposure to noise also has been correlated with cognitive impairment and behavioral problems in children, as well as the more obvious hearing damage and sleep deprivation. The European Environment Agency (EAA) has blamed 900 thousand cases of high blood pressure (hypertension), 43 thousand hospital admissions and 10 thousand cases of premature deaths a year in Europe on noise [21]. Road-traffic noise is the most pervasive noise: 125 million Europeans are exposed to sound pressure levels above 55 decibels (L_den_ 55)—considered as damaging to health. This value is calculated over day, evening and night periods with an emphasis on nighttime. The emphasis on nighttime exposure in L_den_ reflects the importance of sleep. Our data do not directly support this, but it could be concluded that most people are tired and are willing to tweet their dissatisfaction, Figure 1. However, there is a slight bias here to people who are predisposed to tweeting their emotions in a public forum. In addition, the possibility to tweet is only available to people who have access to this App. There may be a gap in the data, which corresponds to people, who work in noisy environments, but do not have access to the App to express their annoyance. Nevertheless, the figure shows that most tweets were reported late at night and in the early morning. Within 2015, 30% of noise annoyance tweets reported equally between October–December and January-March, and 20% equally between April–June and July–September.

Due to network location availability, it was also possible to locate the emirate (in some cases the GPS coordinate in which the tweet was posted and, including the tweet, see Table 3) representing the total number of tweets 74% were in the Emirate of Dubai, 16% in Abu Dhabi, 8% Sharjah and the remaining of an unknown location. Of these, around 70% can be attributed to noise sources located within buildings. Unwanted noise from vehicles and airplanes is usually not categorized as a “noise nuisance”, defined in the UK as “an unlawful interference with a person’s use or enjoyment of land or some right over it, or in connection with it”, [22]. As will be shown, residents tend to be more annoyed by noises that come from uncontrolled human sources (social interaction and increased volume music, Table 4) over predictable, controlled ones (road-traffic).

**Figure 2:**
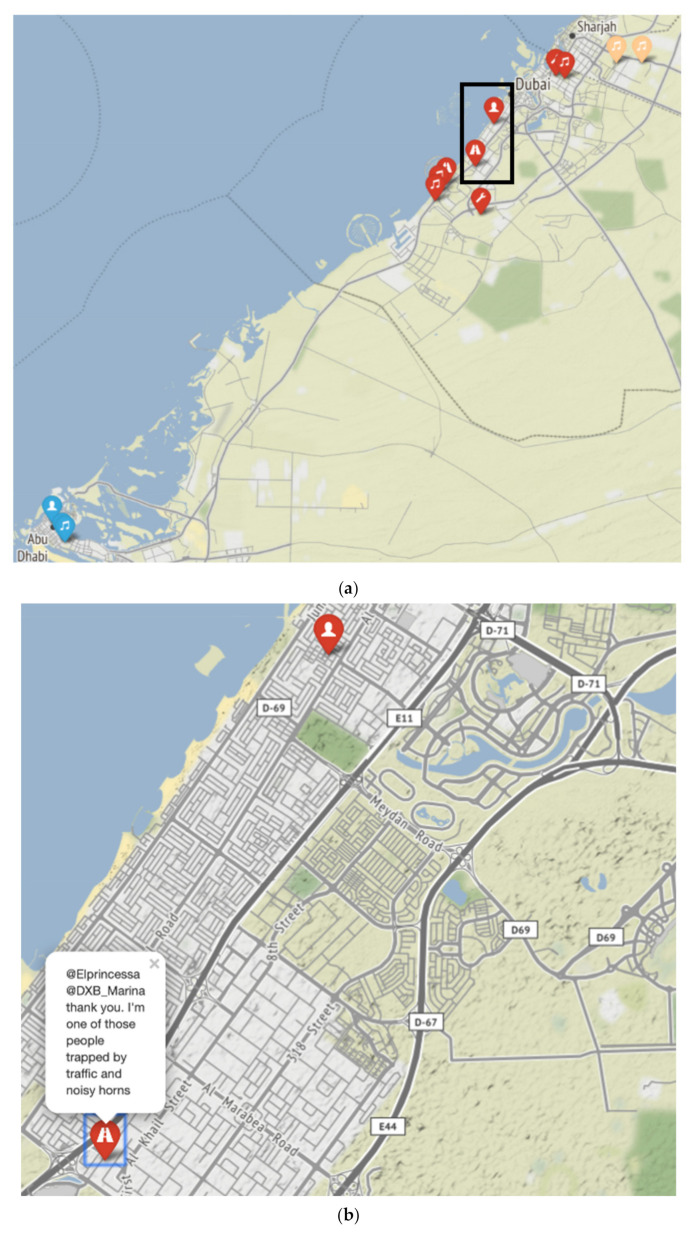

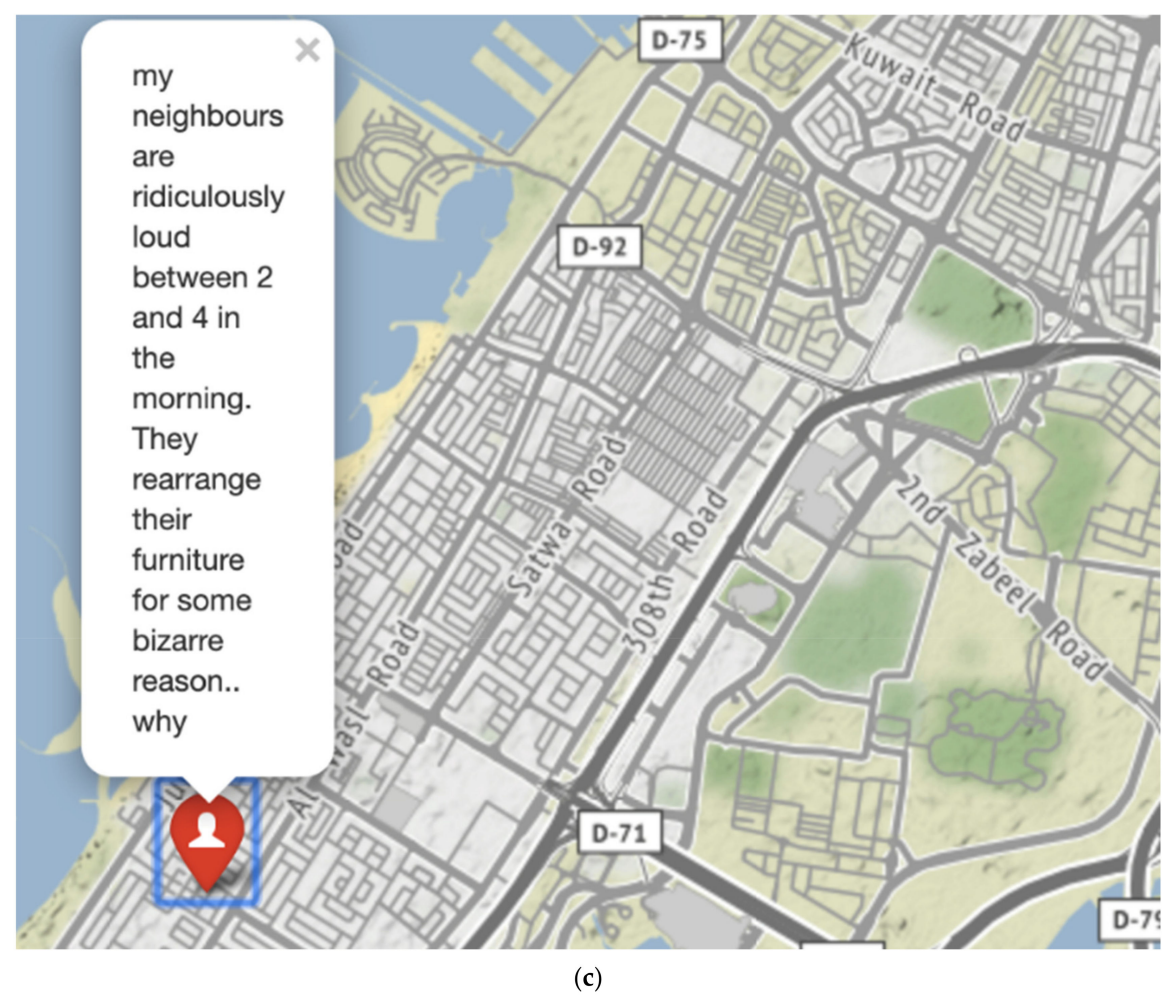
(**a**) Geolocation and characteristic of annoying noises, UAE Twitter 2015, blue = Abu Dhabi, red = Dubai, beige = Sharjah (**b**) reference to insert in (**a**): text “my neighbors are ridiculously loud between 2 and 4 in the morning. They rearrange their furniture for some bizarre reason”, (**c**) text *“@Elprincessa @DXB_Marina thank you. I’m one of those people trapped by traffic and noise horns*”. Legend icons: musical note = music; tool = construction; person silhouette = human; road = traffic; airplane = airplanes; building services = house.

The trend of complaints according to the source type activity is illustrated in Figure 2. Here we can see that music is the most common relative “offender”. This contrasts with conclusions reported by [10] in, which they found degree the highest degree of annoyance was due to aircraft noise (60%), then road traffic (44%), neighborhood exterior (31%), interior (20%), railway (15%) (not applicable to the UAE) and industrial noise (20%).

In this study, Geographic Information System (GIS) technology was utilized to gain some understanding of the spatial distribution and content of a selection of the tweets collected through 2015 according to the source type, Figure 2. It is noted that the most annoying sources, such as music, are in densely populated districts within cities; there appears to be a link between highly populated areas and the frequency of complaints. It should be recognized from municipalities that the number of complaints will rise in these areas as the urban population expands in the UAE.

## 4. Discussion and Conclusions

The use of big data has advantages over other forms of self-reporting in that it captures subjective noise complaints in a relatively naturalistic manner. Big data also has the potential to provide surveillance style reports based on larger datasets spanning multiple years. That said, big data provides a heuristic level of analysis that could form part of a larger, triangulated assessment plan providing cross-validation to objective noise measures and more traditional self-report measures. The representation of sound sources, which were obtained from “tweeting” in social media, including music and neighborhood noise, is affected by several biases since tweeting is an instantaneous reaction to a stimulus. Not all residents have access to social media or immediate access when annoyance occurs. Moreover, many noises are not “available” to be immediately tweeted due to the location of the noise and the presence of the person able to report their findings. This also has a bias on location finding since there could be a large error in the position of the original source. Nevertheless, without the onerous task of manually checking each tweet, it possible to train the query search to accept or decline genuine and accurate data points via machine learning or a knowledge base. Any form of knowledge base could include a larger lexicon than the non-exhaustive example we propose, which could include slang, for example. In the present study, the volume of our dataset resulted in a modest 38 hits, a severe limitation of the team’s present access to the Twitter data set. In future studies with access to a much larger dataset and computer Random Access Memory (RAM) storage availability, perhaps Twitter API open-access data for replicability could be exploited and spanning several years, languages and countries the methods trialed in the present study could prove to be a valuable method for exploring noise pollution and efforts to reduce it.

The present concept study explored the utility of using social media data as a heuristic means of measuring and locating noise pollution trouble spots. This is not to suggest that council services should be employed immediately based on freshly tweeted alarms, but that “annoyance maps” could be created to capture any trends in certain residential districts, for example, which may be noteworthy. Based on the 2015 dataset extracted from the social media platform Twitter, noise annoyance times, locations and sources were identified. Public health statistics worldwide indicate that airport and traffic noise carries the most weight towards medical health problems but targeting and labeling a specific characteristic for noise, which causes the most “annoyance,” is still an open problem. From the small sample extracted in this case study, the data suggest neighborhood or public-entertainment music, not traffic-noise, as the main culprit for “immediate” personal annoyance. Although this study concerns noise, which is an unwanted sound, it could be used in determining areas, which could benefit urban planners or researchers to shape a good “soundscape”.

## Figures and Tables

**Table 1:** Breakdown of language use and unique users from the United Arab Emirates (UAE) Twitter dataset for 2015.

Language	Number of Tweets	Unique Users
Arabic	3,126,163	58,776
English	2,816,777	124,543
Other	2,262,602	6175
Total	8,205,542	189,494

**Table 2:** Lexicon used for filtering Tweets, UAE 2015 (*-represents Wildcard)

#	WORD	AND	AND	OR	OR	OR
1	neighbo(u)r	loud				
2	neighbo(u)r	rowdy				
3	neighbo(u)r	music	annoy *		disturb *	nerves
3	neighbo(u)r	nois *	annoy *		disturb *	nerves
4	music	loud	annoy *	too	disturb *	nerves
5	party	loud	annoy *	too	disturb *	nerves
6	construction	nois *	annoy *	too	sleep *	nerves
7	construction	loud				
8	construction	racket				
9	construction	sleep	annoy *	disturb *		nerves
10	people	shouting	next door	neighbor(u)r	disturb *	nerves
11	people	yelling	next door	neighbor(u)r	disturb *	nerves
12	people	screaming	next door	neighbor(u)r	disturb *	nerves
13	crowd	shouting	next door	neighbor(u)r	disturb *	nerves
14	crowd	yelling	next door	neighbor(u)r	disturb *	nerves
15	crowd	screaming	next door	neighbor(u)r	disturb *	nerves
16	hotel	noise	annoy *	too	disturb *	nerves
17	bar	noise	annoy *	too	disturb *	nerves
18	club	noise	annoy *	too	disturb *	nerves
19	airport	noise	annoy *	too	disturb *	nerves
20	plane	noise	annoy *	too	disturb *	nerves
21	traffic	noise	annoy *	too	disturb *	nerves
22	hotel	loud	too			
23	bar	loud	too			
24	club	loud	too			
25	airport	loud	too			
26	plane	loud	too			
27	traffic	loud	too			
28	traffic	sleep	annoy *	disturb *		nerves
29	jetski	nois *	annoy *	too	disturb *	nerves
30	dog	bark *	annoy *	disturb *		nerves
31	* plane	deafening	annoy *	disturb *		nerves

**Table 3:** Location of annoying noise tweets, UAE 2015.

Emirate	Number of Tweets	Location of Noise Source	Number of Tweets
Dubai	28	Exterior	12
Sharjah	3	Interior	21
Abu Dhabi	6	N/A	5
N/A	1		
Total	38		38

**Table 4:** Types of annoying noises reported by Twitter—UAE 2015.

Annoyance Source Type	#tweets
Music	25
Construction	4
Human	5
Traffic	2
Airplanes	1
Building services (e.g., air conditioning)	1
Total	38

## Data Availability

Data provided by Twitter through commercial agreement.

## References

[1] Freeman C., Francisco L. (2002). As Time Goes By: From the Industrial Revolutions to the Information Revolution.

[2] Ginsberg J., Matthew H., Mohebbi R., Patel S., Brammer L., Smolinski M., Brilliant L. (2009). Detecting influenza epidemics using search engine query data. Nature.

[3] Eysenbach G. (2006). Infodemiology: tracking flu-related searches on the web for syndromic surveillance. AMIA Annual Symposium Proceedings.

[4] Al Shehhi A., Thomas J., Welsch R., Grey I., Aung Z. (2019). Arabia Felix 2.0: a cross-linguistic Twitter analysis of happiness patterns in the United Arab Emirates. J. Big Data.

[5] Dodds P., Harris K., Kloumann I., Bliss C., Danforth C. (2011). Temporal Patterns of Happiness and Information in a Global Social Network: Hedonometrics and Twitter. PLoS ONE.

[6] Chao Y., Srinivasan P. (2016). Life Satisfaction and Pursuit of Happiness on Twitter. PLoS ONE.

[7] Thomas J., Al Shehhi A., Grey I. (2019). The sacred and the profane: social media and temporal patterns of religiosity in the United Arab Emirates. J. Contemp. Relig..

[8] Thomas J., Al-Shehhi A., Al-Ameri M., Grey I. (2019). We tweet Arabic; I tweet English: self-concept, language and social media. Heliyon.

[9] Wenbo W., Chen L., Thirunarayan K., Sheth A. Cursing in English on Twitter. Proceedings of the 17th ACM conference on Computer supported cooperative work & social computing.

[10] Aiello L., Schifanella R., Querica D., Aletta F. (2016). Chatty Maps: constructing sound maps of urban areas from social media data. R. Soc. Open Sci..

[11] Casco L., Clavel C., Asensio C., de Arcas G. (2019). Beyond sound level monitoring: Exploitation of social media to gather citizens subjective response to noise. Sci. Total. Environ..

[12] Gasco L., Schifanella R., Aiello L., Quercia D., Asensio C., de Arcas G. (2020). Social Media and Open Data to Quantify the Effects of Noise on Health. Front. Sustain. Cities.

[13] WHO and JRC Report (2013). Burden of Disease from Environmental Noise. www.euro.who.int/__data/assets/pdf_file/0008/136466/e94888.pdf.

[14] Beutel M., Jünger C., Klein E., Wild P., Lackner K. (2016). Noise Annoyance Is Associated with Depression and Anxiety in the General Population- The Contribution of Aircraft Noise. PLOS ONE.

[15] Passchier-Vermeer W., Passchier W. (2000). Noise Exposure and Public Health. Environ. Health Perspect..

[16] Marianna J., Mariusz W., Konrad L., Grzegorz K. (2017). Noise and environmental pollution from transport: decisive problems in developing ecologically efficient transport systems. J. Vibroeng..

[17] Öhrstrom E., Barregard L., Andersson E., Skanberg A., Svensson H., Angerheim P. (2007). Annoyance due to single and combined sound exposure from railway and road traffic. J. Acoust. Soc. Am..

[18] Selander J., Nilsson M., Bluhm G., Rosenlund M., Lindqvist M., Nise G., Pershagen G. (2009). Long-term exposure to road traffic noise and myocardial infarction. Epidemiology.

[19] Sörensen M., Hvidberg M., Hoffmann B., Andersen Z., Nordsborg R., Lillelund K., Jakobsen J., Tjonneland A., Overvad K., Raaschou-Nielsen O. (2011). Exposure to road traffic and railway noise and associations with blood pressure and self-reported hypertension: a cohort study. Environ. Health.

[20] Theebe M. (2004). Planes, trains, and automobiles: the impact of traffic noise on house prices. J. Real Estate Finance Econ..

[21] Noise in Europe 2014 (2014). EEA Report 10. www.eea.europa.eu/publications/noise-in-europe-2014.

[22] (2004). Environmental Protection Act (UK). https://www.ecolex.org/details/legislation/environmental-protection-act-1990-chapter-43-lex-faoc005515/.

